# Developing in vitro expanded CD45RA^+^ regulatory T cells as an adoptive cell therapy for Crohn's disease

**DOI:** 10.1136/gutjnl-2014-306919

**Published:** 2015-02-24

**Authors:** James B Canavan, Cristiano Scottà, Anna Vossenkämper, Rimma Goldberg, Matthew J Elder, Irit Shoval, Ellen Marks, Emilie Stolarczyk, Jonathan W Lo, Nick Powell, Henrieta Fazekasova, Peter M Irving, Jeremy D Sanderson, Jane K Howard, Simcha Yagel, Behdad Afzali, Thomas T MacDonald, Maria P Hernandez-Fuentes, Nahum Y Shpigel, Giovanna Lombardi, Graham M Lord

**Affiliations:** 1 Medical Research Council Centre for Transplantation, King's College London, London, UK; 2 Department of Experimental Immunobiology, King's College London, London, UK; 3 National Institute for Health Research Biomedical Research Centre at Guy's and St. Thomas’ NHS Foundation Trust and King's College London, London, UK; 4 Department of Gastroenterology, Guy's & St Thomas’ NHS Foundation Trust, London, UK; 5 Department of Immunoregulation and Immune Intervention, King's College London, London, UK; 6 Blizard Institute, Barts and the London School of Medicine and Dentistry, London, UK; 7 The Koret School of Veterinary Medicine, Hebrew University of Jerusalem, Rehovot, Israel; 8 Division of Diabetes and Nutritional Sciences, King's College London, London, UK; 9 Department of Obstetrics & Gynaecology, Hadassah University Hospital, Jerusalem, Israel

**Keywords:** IBD CLINICAL, IMMUNOTHERAPY, INTESTINAL T CELLS, IBD, IBD BASIC RESEARCH

## Abstract

**Background and aim:**

Thymus-derived regulatory T cells (T_regs_) mediate dominant peripheral tolerance and treat experimental colitis. T_regs_ can be expanded from patient blood and were safely used in recent phase 1 studies in graft versus host disease and type 1 diabetes. T_reg_ cell therapy is also conceptually attractive for Crohn's disease (CD). However, barriers exist to this approach. The stability of T_regs_ expanded from Crohn's blood is unknown. The potential for adoptively transferred T_regs_ to express interleukin-17 and exacerbate Crohn's lesions is of concern. Mucosal T cells are resistant to T_reg_-mediated suppression in active CD. The capacity for expanded T_regs_ to home to gut and lymphoid tissue is unknown.

**Methods:**

To define the optimum population for T_reg_ cell therapy in CD, CD4^+^CD25^+^CD127^lo^CD45RA^+^ and CD4^+^CD25^+^CD127^lo^CD45RA^−^ T_reg_ subsets were isolated from patients’ blood and expanded in vitro using a workflow that can be readily transferred to a good manufacturing practice background.

**Results:**

T_regs_ can be expanded from the blood of patients with CD to potential target dose within 22–24 days. Expanded CD45RA^+^ T_regs_ have an epigenetically stable *FOXP3* locus and do not convert to a Th17 phenotype in vitro, in contrast to CD45RA^−^ T_regs_. CD45RA^+^ T_regs_ highly express α_4_β_7_ integrin, CD62L and CC motif receptor 7 (CCR7). CD45RA^+^ T_regs_ also home to human small bowel in a C.B-17 severe combined immune deficiency (SCID) xenotransplant model. Importantly, in vitro expansion enhances the suppressive ability of CD45RA^+^ T_regs_. These cells also suppress activation of lamina propria and mesenteric lymph node lymphocytes isolated from inflamed Crohn's mucosa.

**Conclusions:**

CD4^+^CD25^+^CD127^lo^CD45RA^+^ T_regs_ may be the most appropriate population from which to expand T_regs_ for autologous T_reg_ therapy for CD, paving the way for future clinical trials.

**Significance of this study**

**What is already known on this subject?**

- Thymically derived regulatory T cells (T_regs_) can modulate effector immune responses and, when expanded in vitro, have recently shown promise for graft versus host disease and type 1 diabetes in humans, leading to interest in this therapeutic approach for Crohn's disease.
- Barriers to autologous T_reg_ therapy in Crohn's include the requirement for in vitro expansion to a target dose, potential T_reg_ plasticity to pathogenic interleukin-17^+^ cells, uncertain homing to mucosal tissue and effector T cell resistance to T_reg_-mediated suppression in inflamed Crohn's mucosa.
- Initial enrichment on the basis of CD45RA^+^ expression can improve the phenotypic stability of an expanded T_reg_ population obtained from healthy control blood, but the value of this approach in Crohn's disease is unknown.

**What are the new findings?**

- We show that it is technically feasible to expand functional T_regs_ to numbers consistent with a target dose from the blood of patients with Crohn's disease.
- In vitro expansion enhances the in vitro suppressive activity of these cells. Expanded T_regs_ suppress activation of lamina propria and mesenteric lymph node lymphocytes isolated from inflamed Crohn's mucosa.
- In contrast to T_regs_ expanded from CD45RA^−^ precursors, expanded CD45RA^+^ T_regs_ have epigenetically stable FOXP3 expression and are resistant to Th17 conversion.
- Expanded CD45RA^+^ T_regs_ also express α_4_β_7_ integrin, CD62L and CCR7, and home to human small bowel in a SCID mouse bearing subcutaneously implanted human intestine.

## Introduction

Thymically derived FOXP3^+^ regulatory T cells (T_regs_) are key mediators of peripheral tolerance and are likely to have a role in preventing inappropriate mucosal inflammation in response to bacterial, and other, luminal antigens. In mice, T_reg_ depletion impairs oral tolerance.^1^ Adoptively transferred T_regs_ prevent the onset of colitis or treat established colitis in a number of murine models.^2–7^ *FOXP3* mutations lead to multisystem autoimmunity with enteropathy in mice and humans.^8^ ^9^ Disruption of other key molecules implicated in T_reg_ function, such as transforming growth factor (TGF)-β, Cytotoxic T Lymphocyte-Associated (CTLA)-4, interleukin (IL)-10R subunits, IL-2 or its receptor subunits, is associated with autoimmunity and intestinal inflammation.^10^

Human peripheral blood (PB) or umbilical cord blood T_regs_ can be expanded in vitro through T cell receptor (TCR) stimulation in the presence of IL-2.^11–26^ In vitro expanded human T_regs_ prevent transplant rejection,^27^ ^28^ transplant arteriosclerosis^29^ and graft versus host disease (GvHD)^21^ ^30^ in humanised mice. Promisingly, recent phase 1 clinical trials have shown T_reg_ cell therapy to be safe in patients with GvHD^12^ ^24^ and type 1 diabetes.^18^ Additional phase 1 studies have started in renal (the ONE study) and liver transplantation (ThRIL study).^19^ ^31^

Lamina propria (LP) T_regs_ are increased in the mucosa of patients with active Crohn's disease (CD) and decreased in blood, compared with healthy controls.^32–34^ LP T_regs_ obtained from inflamed CD mucosa suppress proliferation of conventional CD4^+^CD25^lo/int^ T cells (T_con_) obtained from blood but not LP T_cons_,^35^ suggesting that mucosal T_cons_ in active CD may be resistant to T_reg_-mediated suppression. LP T_cons_ from CD mucosa overexpress Smad7, an inhibitor of TGF-β signalling, which confers resistance to T_reg_-mediated suppression.^35^ ^36^ Activated T_cons_ also have an effector-memory phenotype, conferring relative resistance to T_reg_-mediated suppression.^37^ However, T_regs_ expanded in vitro in the presence of rapamycin from the PB of patients with end-stage renal failure (ESRF), systemic lupus erythematosus (SLE), rheumatoid arthritis (RA), multiple sclerosis (MS) and asthma are more suppressive than freshly isolated T_regs_ obtained from the same donor.^26^ ^38^ If it can be shown that in vitro expansion enhances the suppressive ability of CD PB T_regs_ and that these expanded cells suppress mucosal inflammation, parenteral therapy with autologous in vitro expanded T_regs_ generated from CD PB would become a conceptually attractive approach to induce remission in active CD.

IL-17 contributes to mucosal homoeostasis but has also been implicated in the pathogenesis of CD. T_regs_ isolated from healthy donor PB or tonsils can be induced to express IL-17 and the Th17 transcription factor RORC when activated in vitro in the presence of IL-1, IL-2, IL-21 and IL-23.^39–42^ Although major sources of IL-17 in the gut include T_cons_ and γδ T cells, a proportion of T_regs_ obtained from inflamed CD mucosa co-express FOXP3 and IL-17.^43^ Th1 T_reg_ plasticity has also been described in vitro and in vivo.^44^ ^45^ In humans, phenotypically distinct T_reg_ populations can be delineated on the basis of CD45RA expression.^17^ ^46^ ‘Resting’ CD4^+^CD25^hi^CD127^lo^CD45RA^+^ T_regs_ (rT_regs_) are resistant to induction of IL-17 and interferon (IFN)-γ in vitro.^46^ In contrast, ‘activated’ CD4^+^CD25^hi^CD127^lo^CD45RA^−^ T_regs_ (aT_regs_) can be induced to express IL-17 and IFN-γ in vitro.^46^ Similarly, T_regs_ expanded from healthy donor CD45RA^+^ T_regs_ (in the absence of rapamycin) do not contain cytokine producers and are highly suppressive, while T_regs_ expanded from CD45RA^−^ T_regs_ express proinflammatory cytokines and lose *FOXP3* expression with repetitive stimulation in vitro.^17^ ^47^ T_regs_ expanded from healthy control CD45RA^−^ precursors (but not CD45RA^+^ precursors) also have stimulation-induced demethylation of *RORC*, which may be permissive for IL-17 expression.^48^ Furthermore, imprinting α_4_β_7_ integrin expression on in vitro expanded T_regs_ by supplementing culture with all-trans retinoic acid (ATRA) results in high IL-17 expression.^21^ Even though IL-17^+^ T_regs_ isolated from human blood and tonsil retain their suppressive ability in vitro,^39–41^ the potential for adoptively transferred T_regs_ to exacerbate inflammation in CD lesions through the production of proinflammatory cytokines is of significant concern.

Using cell enrichment strategies achievable with currently available good manufacturing practice (GMP) technologies, we show that initial enrichment on the basis of CD45RA^+^ expression is required to generate a homogenous and epigenetically stable T_reg_ population following expansion, in the presence of rapamycin, from the PB of patients with CD. These cells are resistant to Th17 plasticity, express lymphoid and gut homing markers, and home to human gut following adoptive transfer to a SCID mouse bearing subcutaneously implanted human small bowel (SB). In vitro expansion also enhances the suppressive ability of these cells, licensing them to suppress activation of LP and mesenteric lymph node (MLN) T_cons_ obtained from inflamed CD resection specimens. These data suggest that CD PB CD4^+^CD25^hi^CD127^lo^CD45RA^+^ cells may be the most appropriate population from which to expand T_regs_ in vitro for forthcoming clinical trials of autologous T_reg_ cell therapy in CD.

## Materials and methods

### Patient samples

Following Institutional Review Board (IRB) approval (SE London REC 2; 10/H0804/65 and East London REC 2 (10/H0704/74)), patients with CD attending Guy's & St Thomas’ National Health Service (NHS) Foundation Trust and Bart’s Health NHS Trust were invited to donate blood and/or resected tissue. Prospective written consent was obtained. Demographic details are shown in table 1.

**Table 1:** Demographic details of study patients

Female sex	6	(46.1%)
Age (mean±SD)	42.6	(±13.0)
Disease duration	15.4	(±10.4)
Age at diagnosis (mean±SD)	27.7	(±13.1)
Diagnosis <16 years old (A1)	2	(15.4%)
Diagnosis 17–40 years old (A2)	9	(69.2%)
Diagnosis >40 years old (A3)	2	(15.4%)
Location		
Ileal only (L1)	1	(7.7%)
Colonic only (L2)	4	(30.8%)
Ileo-colonic (L3)	8	(61.5%)
Concomitant upper GI disease (L4)	2	(15.4%)
Perianal disease (p)	2	(15.4%)
Behaviour		
Inflammatory (B1)	9	(69.2%)
Stricturing (B2)	2	(15.4%)
Penetrating (B3)	2	(15.4%)
HBI (median, range)	0	(0–7)
Active disease HBI ≥5	4	(30.8%)
Previous surgery	8	(61.5%)
Medications		
Thiopurines	7	(53.8%)
Biologics	4	(30.8%)
Others	3	(23.1%)

GI, gastrointestinal; HBI, Harvey Bradshaw Index.

### T_reg_ enrichment and sorting

Online supplementary figure S1 illustrates the experimental design. Peripheral blood mononuclear cells (PBMCs) were isolated by density gradient centrifugation over lymphocyte separation medium (LSM) 1077 and CD4^+^ lymphocytes enriched to >95% by positive magnetic activated cell separation (MACS) selection (Miltenyi, Bergisch-Gladbach, Germany). Lymphocytes were labelled using the ‘Human Regulatory T Cell Sorting Kit’ (BD Biosciences, San Diego, California, USA), as described previously,^25^ and sorted to CD4^+^CD25^hi^CD127^lo^CD45RA^+^ and CD4^+^CD25^hi^CD127^lo^CD45RA^−^ T_reg_ subsets, and autologous CD4^+^CD25^−^ T_cons_ on a FACSAria (BD; see online supplementary figure S2A–D). Median (IQR) postsort purity was 86.5% (80.8–91.6%; n=13) for CD4^+^CD25^hi^CD127^lo^CD45RA^+^ T_regs_ (CD45RA^+^ T_regs_) and 92.7% (87.7–94.9%; n=13) for CD4^+^CD25^hi^CD127^lo^CD45RA^−^ T_regs_ (CD45RA^−^ T_regs_). Autologous T_cons_ were stored at −80°C.

### In vitro generation of T_reg_ lines

Precursor T_reg_ populations were expanded in vitro as described previously^21^ ^25^ and described in detail in online supplemental methods.

### Cell surface and intracellular stains

Fluorochrome-conjugated antibodies, buffers and experimental technique are listed in online supplemental methods.

### Assessment of the in vitro suppressive ability of putative T_regs_

Assays to determine T_reg_ function in vitro were performed as described previously,^25^ ^49^ and described in detail in the online supplemental methods.

### rtPCR

Following total RNA extraction from Trisure (Bioline, London, UK), cDNA was synthesised using the RevertAid First Strand cDNA Synthesis Kit and multiplex rtPCR performed in duplicate using the Maxima Probe/ROX qPCR Master Mix (both Thermo Fischer Scientific) on a BioRad C1000 Thermal Cycler. Primers are listed in online supplemental methods.

### Estimation of cytokine concentrations

Cytokine concentrations were estimated in culture supernatants using the Cytometric Bead Array (CBA) Human Th1/Th2/Th17 Cytokine Kit (BD) or sandwich ELISAs (R&D), as indicated.

### Assessment of IL-17 production under proinflammatory conditions

In vitro generated T_regs_ were activated with anti-CD3/anti-CD28 beads at a 1:1 ratio and cultured at 10^6^ cells/mL in complete Roswell Park Memorial Institute (RPMI) for 5 days at 37°C/5% CO_2_, supplemented with the following cytokine cocktails, as previously described:^21^ ^23^ ^39^ (A) IL-2 (10 IU/mL, Proleukin); (B) IL-2, IL-1 (10 ng/mL), IL-6 (4 ng/mL) and TGF-β (5 ng/mL); (C) IL-2, IL-21 (25 ng/mL), IL-23 (25 ng/mL) and TGF-β (all R&D Systems). Supernatant IL-17 concentrations were measured by ELISA.

### Assessment of FOXP3 promoter demethylation

Genomic DNA was isolated using a ‘DNeasy kit’ (Qiagen, Manchester, UK). Bisulfite conversion and assessment of the methylation status of the *FOXP3* T_reg_-specific demethylated region (TSDR) was performed by Epiontis.^50^ ^51^ The genomic locations of *FOXP3* and *GAPDH* CpG-rich regions probed have been reported.^51^

### Isolation of LP mononuclear cells and MLN mononuclear cells

LP mononuclear cells (LPMCs) and MLN mononuclear cells (MLNMCs) were isolated as described previously^52^ and listed in online supplemental methods.

### C.B-17 SCID mouse human intestinal xenotransplant model

Experimental design is illustrated in figure 3C. The C.B-17 SCID mouse human intestinal xenotransplant model has been described previously^53^ ^54^ and is described in detail in online supplemental methods. IRB and IACUC approvals were obtained prospectively (Ethics Committee for Animal Experimentation, Hebrew University of Jerusalem; MD-11-12692-4 and the Helsinki Committee of the Hadassah University Hospital; 81-23/04/04). Techniques for the detection of adoptively transferred T_regs_ are also described in detail in online supplemental methods.

### Statistical analysis

Statistical analysis was carried out using GraphPad Prism 5 (GraphPad Software Inc, La Jolla, California, USA) and the methods used are described in detail in the online supplemental methods.

## Results

### T_regs_ can be expanded from the blood of patient with CD using GMP-compatible protocols

Hoffmann *et al*^17^ showed that initial T_reg_ enrichment on the basis of CD45RA^+^ expression was required to expand homogenous and stable T_reg_ lines from healthy donors in the absence of supplemental rapamycin. Rapamycin prevents the outgrowth of contaminating T_cons_ in T_reg_ cultures, and may make the requirements for the starting population less stringent.^11^ ^13^ ^15^ ^21^ ^23^ ^55^ However, the optimum precursor population from which to expand a homogenous, suppressive and epigenetically stable T_reg_ population from CD PB is currently unknown. In previous studies, we accomplished in vitro expansion of in vitro suppressive T_regs_ from healthy controls^21^ and renal transplant candidates.^26^ We sought to determine if T_regs_ could be expanded in vitro from the blood of patients with CD.

Freshly isolated CD4^+^ lymphocytes from 13 patients with CD were fluorescence-activated cell sorting (FACS)-sorted into CD4^+^CD25^hi^CD127^lo^CD45RA^+^ (median (IQR) of 2200 cells/mL PB (860–4400)) and CD4^+^CD25^hi^CD127^lo^CD45RA^−^ subsets (3700 cells/mL (2000–4500)), then expanded in vitro in the presence of high-dose IL-2, rapamycin and anti-CD3/anti-CD28 beads. Active disease, evidenced by a Harvey Bradshaw Index >5 (n=4) *or* elevated C reactive protein (n=1), was not associated with a significantly reduced yield (see online supplementary figure S2E). Donor clinical characteristics are given in table 1.

**Figure 1:**
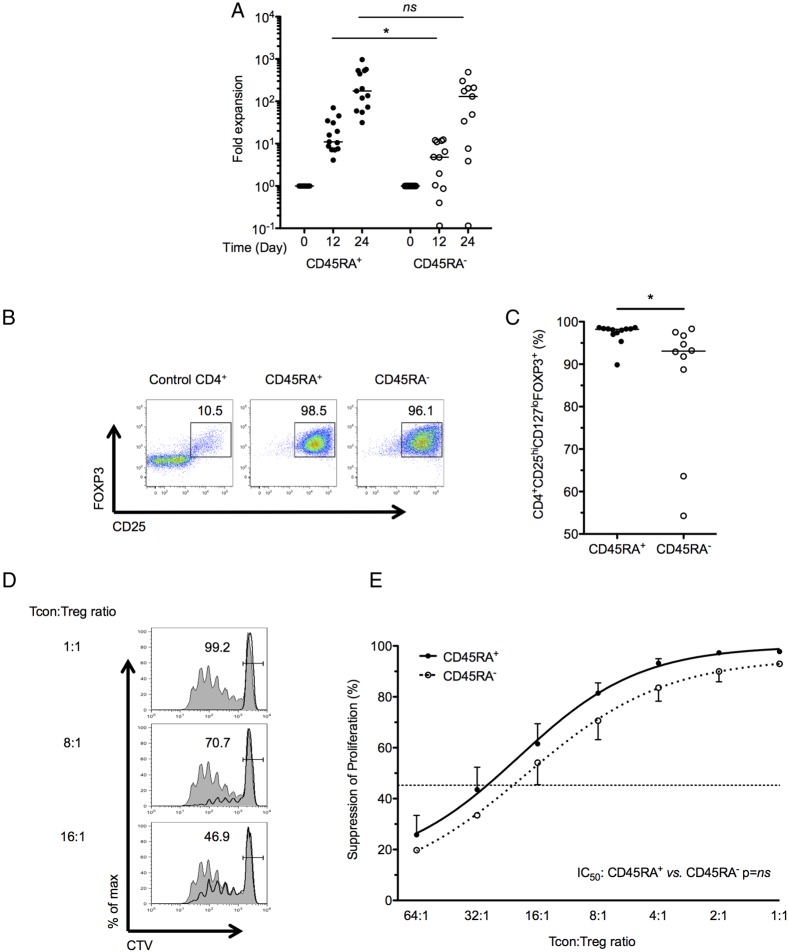
Expansion, phenotype and potency of in vitro expanded T_regs_. (A) Cumulative fold expansion of T_reg_ lines at days 12 and 24 of culture, grouped according to CD4^+^CD25^hi^CD127^lo^CD45RA^+^ or CD4^+^CD25^hi^CD127^lo^CD45RA^−^ precursors; n=13 each, bar: median. (B) Representative FACS plots gated on live events showing CD25 and FOXP3 expression at D24. (C) Proportion of T_regs_ with a CD4^+^CD25^hi^CD127^lo^ T_reg_ phenotype at D24. (D) Representative plots from a proliferation assay, illustrating dose-dependent suppression of T_con_ proliferation by CD45RA^+^ T_regs_. Proliferation CTV-labelled autologous CD4^+^CD25^−^ T_cons_ alone (filled) or with T_regs_ at various T_con_:T_reg_ ratios (bold line) is shown. (E) D24 T_reg_-mediated suppression of T_con_ proliferation. Cumulative data showing mean±SEM suppression seen at each T_con_:T_reg_ ratio. Pooled data from 29 T_reg_ lines. Comparisons between suppression seen in study conditions and mean non-specific suppression seen in ‘2X’ control condition (dotted line) are shown. *p<0.05,***p<0.001 and ****p<0.0001. T_regs_, thymus-derived regulatory T cells; FACS, fluorescence-activated cell sorting; T_cons_, conventional CD4^+^CD25^lo/int^ T cells; CTV, Cell Trace Violet; NS, not significant.

Every CD45RA^+^ T_reg_ line proliferated, to a median (IQR) of 175-fold (66–531; n=13) at D24 (figure 1A). In contrast, 3 of 13 (23%) CD45RA^−^ T_reg_ lines did not proliferate and were discontinued. CD45RA^−^ T_regs_ expanded 130-fold (8–209; n=10). Expanded T_regs_ were exclusively CD4^+^ lymphocytes. Expression of CD25 and FOXP3 was comparable in D24 CD45RA^+^ and CD45RA^−^ T_regs_ (figure 1B), but a greater proportion of CD45RA^+^ T_regs_ maintained a CD4^+^CD25^hi^CD127^lo^FOXP3^+^ phenotype (p=0.037; figure 1C).

Proliferation assays were performed to determine if in vitro expanded T_regs_ retained the ability to suppress proliferation of autologous CD4^+^CD25^−^ T_cons_. CD45RA^+^ and CD45RA^−^ T_regs_ suppressed T_con_ proliferation to an equivalent degree (figure 1D–E), demonstrating specific suppression (vs the 2X cell density control) above an 8:1 T_con_:T_reg_ ratio. CD45RA^+^ and CD45RA^−^ T_regs_ reduced IL-2 expression in 96 h co-culture supernatants (see online supplementary figure S3A). CD45RA^+^ T_regs_ also suppressed IFN-γ expression in 96 h co-culture supernatants (see online supplementary figure S3B).

### In vitro expanded CD45RA^+^ T_regs_ are resistant to IL-17 induction and stably express FOXP3

**Figure 2:**
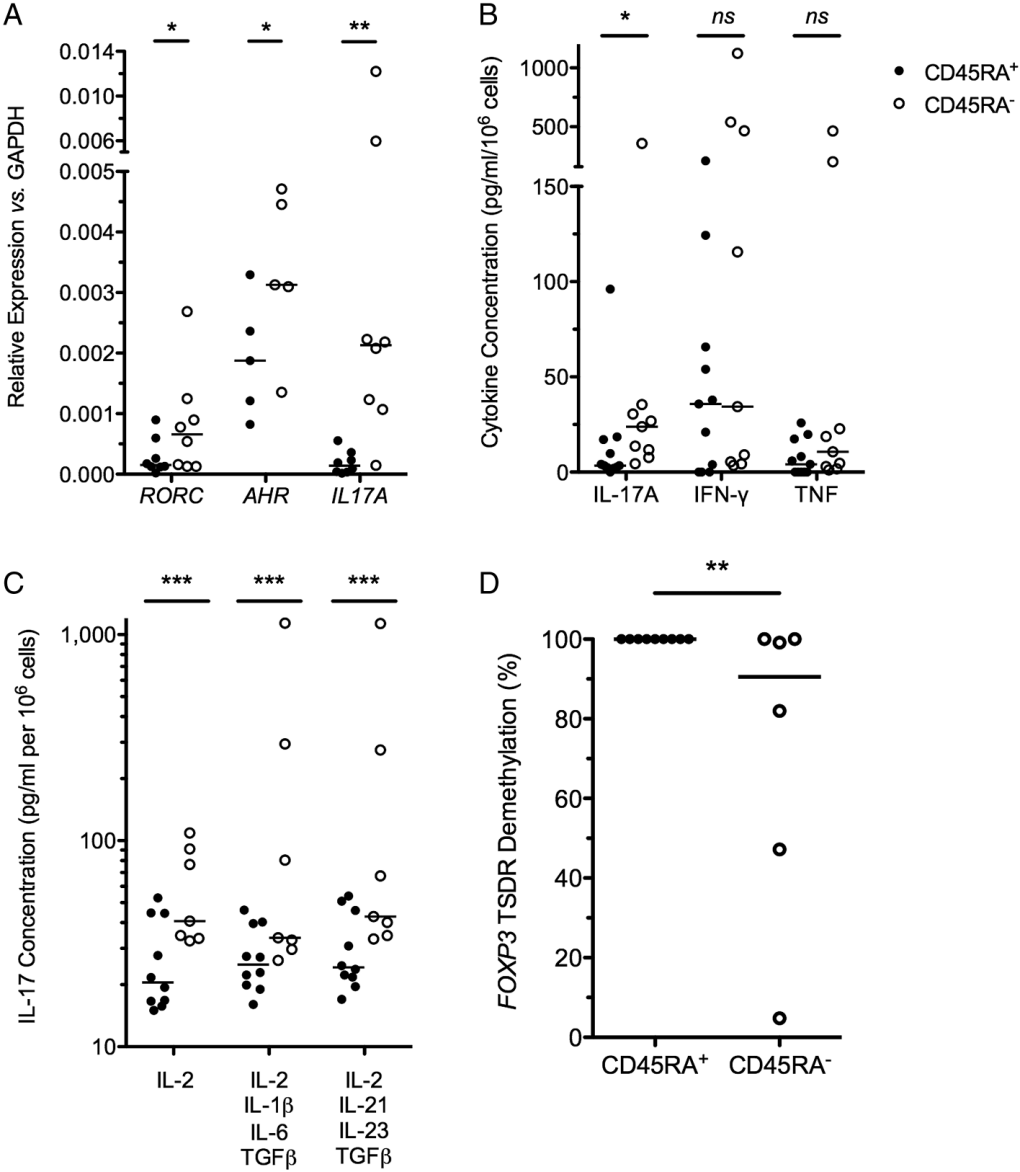
D24 CD45RA^+^ T_regs_ are resistant to IL-17 induction. (A) Relative expression of *IL17A*, *RORC* and *AHR* in D24 CD45RA^+^ and CD45RA^−^ T_regs_, relative to *GAPDH*; n=16, bar at median. (B) D24 T_reg_ IL-17, IFN-γ and TNF secretion in 24 h culture supernatants; n=20, bar at median. (C) IL-17 detected by ELISA from 5-day culture supernatants of D24 T_regs_ cultured in the absence of rapamycin but with supplemental IL-2 alone, a cocktail of IL-2, IL-1, IL-6 and TGF-β or a cocktail of IL-2, IL-21, IL-23 and TGF-β. n=17, bar at median. (D) % FOXP3 TSDR demethylation; n=15, bar at median. *p<0.05,**p<0.01,***p<0.001. T_regs_, thymus-derived regulatory T cells; IL, interleukin; IFN, interferon; TNF, tumour necrosis factor; TGF, transforming growth factor; TSDR, T_reg_-specific demethylated region; NS, not significant.

The ‘inflammatory potential’ of in vitro expanded T_regs_ from patients with CD was examined. Genes important in Th17 biology, including *RORC*, *AHR* and *IL-17*, were significantly overexpressed in CD45RA^−^ T_regs_, in comparison with expression in paired CD45RA^+^ T_regs_ (p<0.05 for each comparison, figure 2A). IL-17 secretion was also significantly different in these T_reg_ subsets. IL-17 expression was below the limit of detection in 10/11 (91%) CD45RA^+^ T_regs_ and significantly higher in CD45RA^−^ T_regs_ (p=0.02; figure 2B).

The potential of in vitro expanded T_regs_ to turn on an inflammatory programme following exposure to Th17-inducing cytokines, as occurs in vitro in T_regs_ isolated from blood,^39–41^ was examined. D24 T_regs_ were washed and cultured for a further 5 days in the presence of IL-2 alone, or Th17-inducing cytokines (IL-2, IL-1, IL-6 and TGF-β or IL-2, IL-21, IL-23 and TGF-β; figure 2C). These proinflammatory cytokines failed to induce IL-17 production by CD45RA^+^ T_regs_. In contrast, IL-17 production by CD45RA^−^ T_regs_ was 3-fold higher than CD45RA^+^ T_regs_ in neutral conditions (IL-2 alone) and 10-fold higher in skewing conditions (p<0.001 each comparison).

To ensure that phenotypic stability of CD45RA^+^ T_regs_ correlated with an epigenetically stable *FOXP3* locus, we determined the methylation status of the *FOXP3* ‘TSDR’ (figure 2D). We found the TSDR to be completely demethylated in all CD45RA^+^ T_reg_ lines tested (100%; n=9), suggesting an epigenetically stable FOXP3 locus in CD45RA^+^ T_regs_ even after 24d of in vitro expansion. In contrast, variable degrees of TSDR demethylation were seen in CD45RA^−^ T_reg_ lines (median (IQR) of 90.6% (36.6%–100%); n=6; p=0.008).

### In vitro expanded CD45RA^+^ T_regs_ express homing receptors for gut and lymphoid tissue

**Figure 3:**
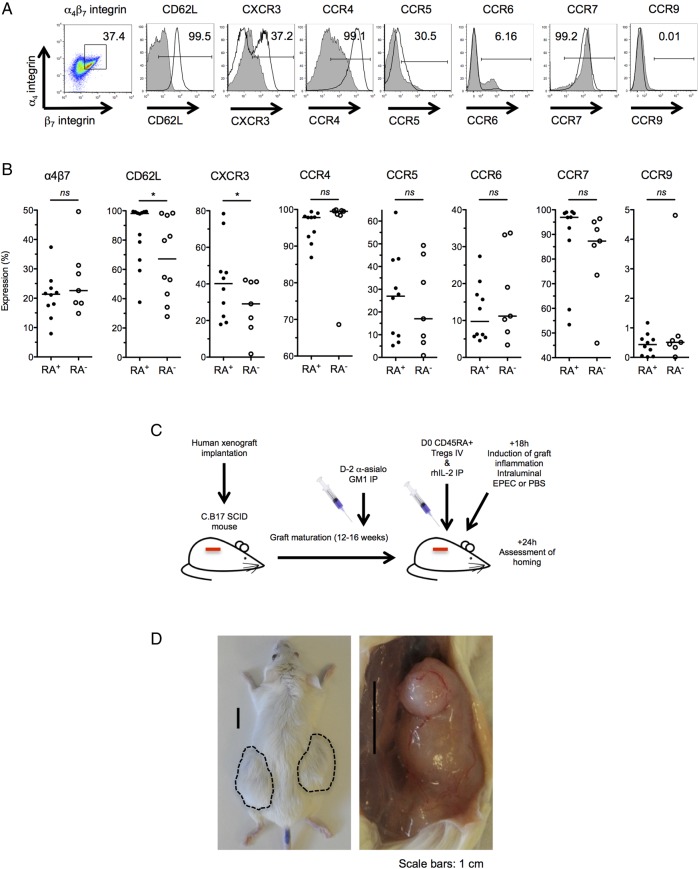

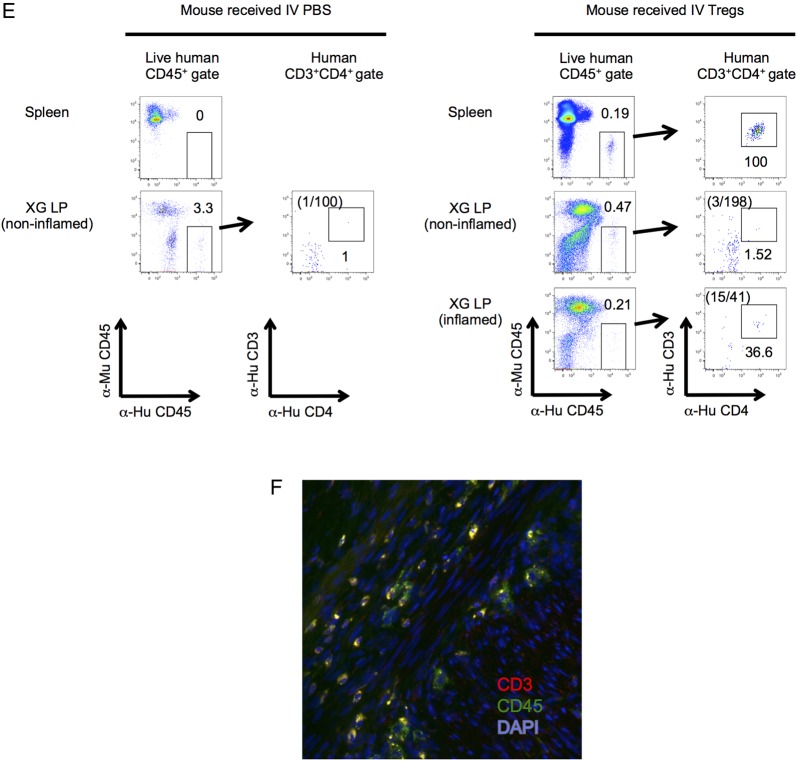
D24 CD45RA^+^ T_regs_ express gut and lymphoid homing receptors and home to inflamed human LP in a C.B-17 severe combined immunodeficiency (SCID) mouse human intestinal XG model. (A) Representative FACS plots illustrating gut and lymphoid homing receptor expression on D24 CD45RA^+^ T_regs_ (bold line). Gates were drawn on the basis of fully stained CD4^+^ lymphocytes (filled) and fluorochrome minus one (FMO) controls. (B) Dot plots showing expression of intestinal and lymphoid homing receptors in D24 CD45RA^+^ and CD45RA^−^ T_regs_. n=17; *p<0.05. (C) Design of the XG mouse experiment. (D) Left panel: mature XGs (circled) are visible subcutaneously on the dorsum of the mouse. Right panel: dorsal skin has been removed in an anaesthetised mouse to reveal the mucus-filled XG in situ (right panel). Microscopic images of the XG are shown in online supplementary figure S4A. (E) FACS plots showing live human CD45^+^CD3^+^CD4^+^ events in single cell suspensions prepared from murine spleen, non-inflamed and inflamed XGs, 24 h after intravenous phosphate buffered saline (PBS) (left panels) or adoptive transfer of T_regs_ (right panels). The absolute numbers of CD3^+^CD4^+^ events in the XG human CD45^+^ gates are highlighted. The gating strategy is illustrated in online supplementary figure S4B. (F) Immunofluorescence staining of XG cryosections with antihuman CD3 (red), antihuman CD45 (green) and 4′,6-diamidino-2-phenylindole (DAPI) (blue). (E and F) Representative of two independent experiments. T_regs_, thymus-derived regulatory T cells; LP, lamina propria; XG, xenograft; FACS, fluorescence-activated cell sorting; EPEC, enteropathogenic *Escherichia coli*; NS, not significant.

The ability of in vitro expanded T_regs_ to home to relevant immune niches, where they may suppress inflammation, is thought to be critical for cell therapy. Consequently, the expression of gut homing receptors on in vitro expanded T_regs_ was examined by FACS (figure 3A, B). We found that D24 CD45RA^+^ T_regs_ modestly expressed α_4_β_7_ integrin and CCR6 (20.8%±7.8% and 12.2%±7.9%, respectively) and did not express CCR9. Both CD62L (84.8%±20.6%; p=0.04 vs CD45RA^−^) and CCR7 (92.1%±12.8%; p=0.03) required for lymph node homing were more highly expressed in CD45RA^+^ T_regs_ than CD45RA^−^ T_regs_. CCR4 (95.4%±4.2%) was also highly expressed.

### Adoptively transferred CD45RA^+^ T_regs_ home to inflamed human small intestine in a C.B-17 SCID human SB xenotransplant model

In view of the favourable phenotype of CD45RA^+^ T_regs_ as a candidate cell therapy, we next sought to determine whether these cells could home to inflamed human SB in vivo. D24 CD45RA^+^ T_regs_ were administered to a C.B-17 SCID mouse bearing human small intestinal xenotransplants and homing assessed 24 h later (figure 3C, D). Intraluminal injection with enteropathogenic *Escherichia coli* was used to induce mucosal inflammation (see online supplementary figure S4A). Following adoptive transfer, human CD45^+^CD3^+^CD4^+^ cells were detected in mouse spleen and inflamed human SB LP by FACS (see figure 3E; gating strategy online supplementary figure S4B), indicating that adoptively transferred CD45RA^+^ T_regs_ homed to inflamed human SB LP in this model. This was confirmed by the detection of human CD45^+^CD3^+^ cells in inflamed human SB LP by immunofluorescence (figure 3F). We previously showed that human fetal SB contains a population of CD3^−^CD7^+^ cells that persist following xenotransplantation.^53^ Human CD45^+^CD3^−^ events were also detected in non-inflamed human SB LP in both mice that received intravenous PBS and intravenous T_regs_ (figure 3E), suggesting that a population of long-lived human immune cells was co-transferred with the human SB transplant.

### In vitro expansion enhances the in vitro suppressive ability of CD45RA^+^ T_regs_

**Figure 4:**
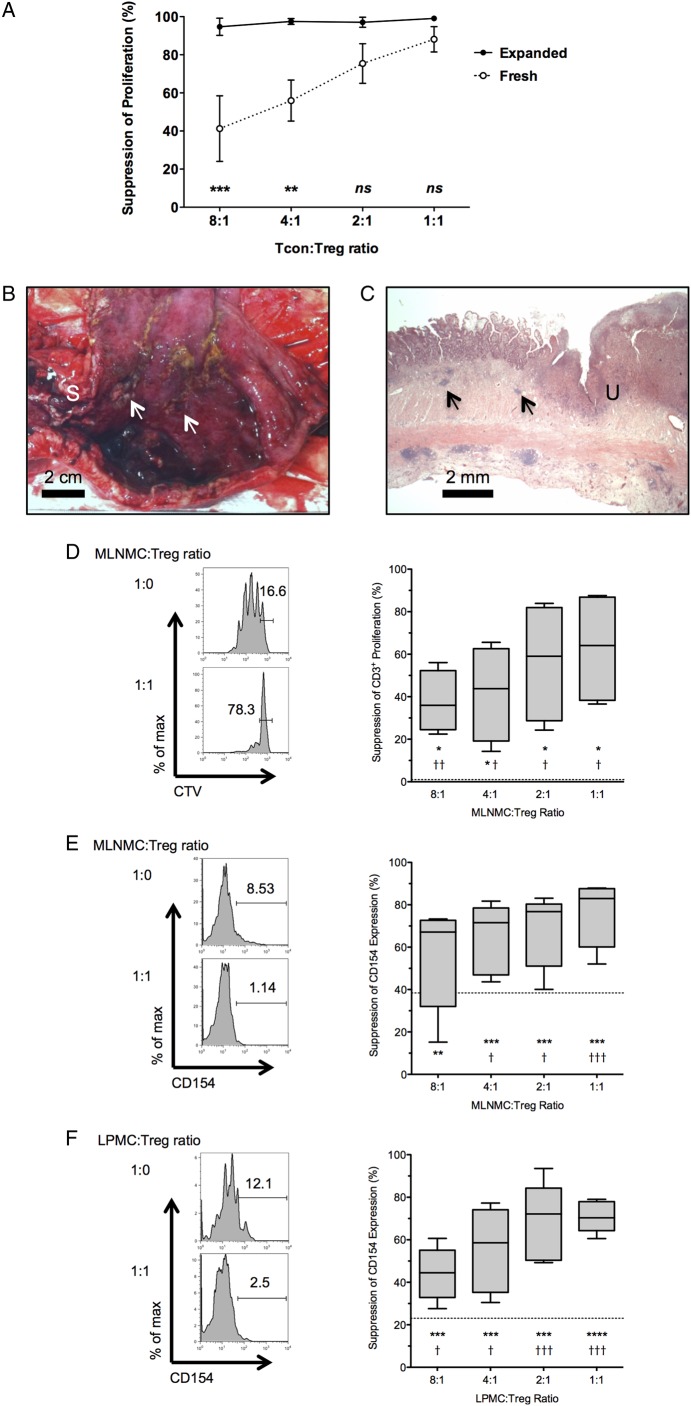
In vitro expanded CD45RA^+^ T_regs_ suppress CD3^+^ T cell responses from inflamed Crohn's MLN and LP. (A) Suppression of proliferation of a single lot of freeze-thawed, allogeneic T_cons_ by freshly isolated PB CD4^+^CD25^hi^CD127^lo^CD45RA^+^ T_regs_, or D24 CD45RA^+^ T_regs_ that were expanded in vitro from these freshly isolated precursors. Pooled data from three sets of freshly isolated PB T_regs_ and subsequently expanded T_reg_ populations. Data points are mean±SEM. (B) Fresh ileal resection specimen opened longitudinally to show ileal stricture (marked ‘S’) and proximal inflamed, haemorrhagic mucosa with deep ulceration (arrows). Scale bar: 2 cm. (C) Representative microscopic image from this resection showing mucosal distortion, ulceration (marked ‘U’) and transmural inflammation, including lymphoid aggregates (arrows). 12.5× H&E. Scale bar: 2 mm. (D) Representative FACS plots gated on live CD3^+^ events, showing proliferation of MLN T_cons_ cultured alone (top left panel) or with T_regs_ at a 1:1 MLNMC:T_reg_ ratio (bottom left panel). Pooled data showing T_reg_-mediated suppression of MLN CD3^+^ proliferation (right panel, n=5). Box and whisker plot shows median, IQR and range. (E) Representative FACS plots gated on live MLN CD3^+^ events showing CD154 expression on MLN T_cons_ cultured alone (top left panel) or with T_regs_ at a 1:1 MLNMC:T_reg_ ratio (bottom left panel). Pooled data showing T_reg_-mediated suppression of CD154 expression in live MLN CD3^+^ cells (right panel, n=5). (F) Representative FACS plots gated on live LP CD3^+^ events showing CD154 expression on LP T_cons_ cultured alone (top left panel) or with T_regs_ at a 1:1 LPMC:T_reg_ ratio (bottom left panel). Pooled data showing T_reg_-mediated suppression of CD154 expression in live LP CD3^+^ cells (right panel, n=5). (D–F) Dotted line shows non-specific suppression from ‘2X control’. Comparisons between observed suppression and non-specific suppression (†p<0.05, ††p<0.01, †††p<0.001, ††††p<0.0001) and observed suppression and no suppression (zero, *p<0.05, **p<0.01, ***p<0.001 and ****p<0.0001) are shown. T_regs_, thymus-derived regulatory T cells; MLN, Mesenteric lymph node; LP, lamina propria; T_cons_, conventional CD4^+^CD25^lo/int^ T cells; PB, peripheral blood; FACS, fluorescence-activated cell sorting; MLNMC, MLN mononuclear cell; LPMC, LP mononuclear cell; CTV, Cell Trace Violet.

LP T_cons_ from inflamed CD mucosa are resistant to in vitro suppression by autologous LP T_regs._^35^ ^36^ Consequently, it is possible that in vitro expanded T_regs_ will need an enhanced suppressive function in order to be successful as a future cell-based therapy. Expansion with supplemental rapamycin enhances the in vitro suppressive ability of T_regs_ from patients with ESRF, SLE, RA, MS and asthma.^26^ ^38^ In order to determine if in vitro expansion enhanced T_reg_ function in patients with CD, freshly isolated CD4^+^CD25^hi^CD127^lo^CD45RA^+^ T_regs_ or D24 CD45RA^+^ T_regs_ that were expanded in vitro from these FACS-sorted CD4^+^CD25^hi^CD127^lo^CD45RA^+^ precursors were co-cultured with allogeneic Carboxyfluorescein succinimidyl ester (CFSE)-labelled CD4^+^CD25^−^ T_cons_ (n=3 independent experiments; cells from the same lot of single-donor, freeze-thawed T_cons_ for each experiment). D24 CD45RA^+^ T_regs_ suppressed T_con_ proliferation to a greater degree than the freshly isolated CD4^+^CD25^hi^CD127^lo^CD45RA^+^ T_regs_ from which they were expanded, at both a 4:1 and 8:1 T_con_:T_reg_ ratio (p<0.01 and p<0.001, respectively; figure 4A). This suggests that in vitro expansion enhances the suppressive ability of D24 CD45RA^+^ T_regs_.

### In vitro expanded CD45RA^+^ T_regs_ suppress proliferation and activation of MLN and LP T cells in active CD

We next wished to determine if D24 CD45RA^+^ T_regs_ could suppress activation and proliferation of T_cons_ taken from the MLN and LP of patients with CD (figure 4B, C). MLNMCs were co-cultured with T_regs_ and CD3^+^ proliferation assessed at 96 h. Dose-dependent T_reg_-mediated suppression of MLN CD3^+^ proliferation was seen at each MLNMC:T_reg_ ratio (figure 4D). We were unable to demonstrate in vitro suppression of LPMC CD3^+^ proliferation with this technique, as both freshly isolated and freeze-thawed LPMCs obtained from inflamed CD mucosa died prior to acquisition at 96 h (n=4 independent experiments; see online supplementary figures S5 and S6).

We recently validated a novel co-culture assay for the assessment of in vitro expanded T_reg_ function. This takes advantage of T_reg_-mediated suppression of the early activation marker CD154 (CD40 L) on T_cons_ at 7 h, which correlates with T_reg_-mediated suppression of CFSE dilution and cytokine expression in T_cons_ at 96h^25^ ^49^ Significant dose-dependent suppression of CD154 expression in MLN and LP T cells was observed (figure 4E, F), demonstrating that in vitro expanded D24 CD45RA^+^ T_regs_ suppress early activation of MLN and LP T_cons_ in vitro.

## Discussion

There remains an unmet need to develop novel therapies for CD, as current drug treatments frequently fail to maintain long-term remission and may be complicated by significant side effects. Cellular therapies are emerging as potentially attractive therapeutic strategies. T_regs_ are effective in preclinical models of colitis^2^ ^6^ and phase 1 clinical trials suggest that in vitro expanded T_regs_ are safe in the prophylaxis and treatment of GvHD^12^ ^24^ and type 1 diabetes.^18^ We built on recent work to describe a method for isolation and expansion of T_regs_ from Crohn's blood that is readily transferable to a GMP background and addresses several barriers to the use of expanded T_regs_ as an autologous cell-based therapy in this important disease.

T_regs_ can be selected and expanded in vitro to clinically useful numbers under both R&D-grade,^11 13^ ^16^ ^21^ ^23^ ^26^ and GMP conditions^12^ ^18^ ^24^ retaining an in vitro suppressive function before infusion into humans. We showed that it is feasible to do the same using T_regs_ obtained from Crohn's blood, including patients receiving thiopurines or anti-tumor necrosis factor (TNF) medications. Even after prolonged culture, these T_regs_ maintained FOXP3 expression and suppressed activation of autologous T cells.

T cell lineage plasticity is well described. A major potential barrier to T_reg_ therapy is the possibility that these cells might adopt an inflammatory phenotype and worsen inflammation on adoptive transfer. Freshly isolated thymus-derived T_regs_ from both mice and humans can express proinflammatory cytokines and transcription factors (TF) canonical to effector CD4^+^ lineages, including IL-17^39–41^ and IFN-γ,^44^ both of which are implicated in CD pathogenesis. Indeed, IL-17^+^FOXP3^+^ T_regs_ have been identified in non-inflamed human blood and lymphoid tissue,^40^ and inflamed Crohn's mucosa.^43^ While there is some evidence that plastic cytokine and TF expression may license efficient T_reg_ homing to, and suppression of, Th1-mediated and Th17-mediated inflammation,^44^ ^56^ this may also lead to the generation of T_regs_ with an effector phenotype that contribute to inflammation.

We and others have demonstrated that in vitro expanded T_regs_ cultured in the presence of rapamycin have enhanced phenotypic stability.^13^ ^21^ We show that as well as retaining their suppressive capacity, CD45RA^+^ rT_regs_ expanded from the blood of patients with CD in the presence of rapamycin do not express IL-17A or other Th17-related genes, even following exposure to proinflammatory cytokines that they would likely meet in inflamed intestinal mucosa. These data corroborate data from Hoffmann *et al*^17^ ^47^ in healthy controls, showing that expanded CD45RA^+^ T_regs_ are resistant to the induction of proinflammatory cytokines on stimulation and highly express CD62L and CCR7, which are associated with phenotypic stability.

Freshly isolated CD45RA^+^ rT_regs_ have an epigenetically stable *FOXP3* locus with extensive TSDR demethylation.^46^ TSDR demethylation correlates with stable FOXP3 expression in vitro^50^ and T_reg_-mediated protection from autoimmunity in vivo^57^ in humans. However, the significance of TSDR demethylation for in vitro expanded T_regs_ is poorly understood. Barzaghi *et al*^57^ recently described a cohort of patients with ‘Immune dysregulation, Polyendocrinopathy, Enteropathy, X-linked syndrome (IPEX)-like syndrome’, severe multisystem autoimmunity in the absence of identifiable mutations in molecules implicated in T_reg_ function, with decreased TSDR demethylation despite normal T_reg_ numbers and in vitro suppression. This suggests that ex vivo expanded CD45RA^−^ T_regs_, with incomplete TSDR demethylation, may have suboptimal biological activity in vivo, despite suppressive function in vitro. These data also suggest that CD45RA^+^ T_regs_ are more likely to retain phenotypic stability and are less likely to acquire an effector phenotype than CD45RA^−^ T_regs_, consistent with a more favourable safety profile of this T_reg_ subset as a cell-based therapy for CD.

In order to be therapeutically effective, adoptively transferred T_regs_ may need to traffic to intestinal lymphoid tissue or LP. Some groups have taken advantage of TCRs specific for luminal antigens to direct T_regs_ to the intestinal mucosa, such as IL-10-producing T cell clones with ovalbumin-specific TCRs,^58^ or T cells with transgenic Cbir1 flagellin-specific TCRs.^59^ Alternatively, T_reg_ expansion in the presence of ATRA induces α_4_β_7_ integrin expression but also increases effector cytokine expression, such as IL-17 and IFN-γ, potentially limiting its use in GMP cell expansion.^13^ ^21^ We show that CD45RA^+^ T_regs_ expanded in the presence of IL-2 and rapamycin highly express CD62L and CCR7, allowing homing to, and anatomical orientation within lymphoid tissue.^60^ ^61^ T_reg_ CD62L expression is also required for T_reg_-mediated cure of GvHD.^30^ CD45RA^+^ T_regs_ also expressed CCR4, required for T_reg_-mediated prevention of CD45RB^hi^ colitis.^62^ Interestingly, murine T_regs_ do not need to home to intestinal LP to prevent CD45RB^hi^ adoptive transfer colitis. β_7_ integrin-null T_regs_ home to MLN and prevent colitis in this model, despite almost undetectable LP homing.^63^ Consequently, the ability to home to MLN is highly desirable in potentially therapeutic cells.

CD45RA^+^ T_regs_ also express α_4_β_7_ integrin and CXC motif receptor 3 (CXCR3), indicating an ability to home to LP and sites of inflammation, respectively. Moreover, we used a human small intestinal xenotransplant model to show, for the first time, that in vitro expanded CD45RA^+^ T_regs_ from patients with CD home to inflamed human gut in vivo. Xenotransplanted SB segments develop into tissue that is morphologically and functionally identical to normal gut and is capable of peristalsis and nutrient absorption.^53^ ^54^ The xenografts also possess a chimeric endothelium that expresses human MadCAM-1.^64^ This is the first demonstration that this model can be used in the assessment of immune cell homing.

Xenograft-bearing mice received rhIL-2 in order to support survival of adoptively transferred human T_regs_,^23^ as murine IL-2 is less efficient at promoting proliferation of human T cells than rhIL-2, despite cross-reactivity.^65^ As recent phase 1 trials of in vitro expanded T_regs_ in GvHD and type 1 diabetes mellitus showed signs of clinical efficacy without supplemental rhIL-2, it is likely that this is a feature of the experimental system and will not be required in clinical trials in Inflammatory bowel disease (IBD).^12^ ^18^ ^24^

Future work will include ‘humanising’ xenograft-bearing mice and developing additional techniques to induce xenograft inflammation, thus allowing us to assess the functional impact of CD45RA^+^ T_regs_ on gut inflammation. The percentage of LP human T cells that could be recovered from human bowel transplants was relatively modest compared with the percentage of T cells recovered from the spleen. Given that the expression of the gut homing integrin α_4_β_7_ was only expressed on ∼20% of the purified T_regs_, future work may need to address methods to increase α_4_β_7_ expression, such as the use of retinoic acid, as we have previously shown.^21^

An additional barrier to T_reg_ therapy in CD is that effector T cells from the diseased mucosa of patients with CD may be resistant to the suppressive action of T_regs_. Indeed, we previously showed that T_cons_ isolated from inflamed Crohn's mucosa are relatively resistant to T_reg_-mediated suppression, due to overexpression of Smad7, an inhibitor of TGF-β signalling.^35^ ^36^ In this study, we utilised T_regs_ cultured in the presence of rapamycin, which has been shown to enhance the suppressive ability of in vitro expanded T_regs_, compared with T_regs_ freshly isolated from the same donor^26^ ^38^ and show that in vitro expansion enhances the suppressive ability of T_regs_ obtained from CD PB. Rapamycin-expanded CD45RA^+^ T_regs_ effectively suppress both MLN and LP T cells obtained from inflamed Crohn's resection specimens. These data suggest that in vitro expanded CD45RA^+^ T_regs_ may modulate immune responses in niches directly relevant to the pathogenesis of CD. T_regs_ use multiple mechanisms to suppress in vitro and in vivo, including contact-dependent mechanisms (CTLA-4, perforin-granzyme B) and contact-independent mechanisms (IL-10, TGF-β, extracellular ATPase activity via CD39/CD73,etc). Sakaguchi *et al*^10^ has proposed a multistep model of in vitro suppression that initially requires cell-cell contact but is subsequently contact independent. The mechanism of suppression of erstwhile ‘resistant’ mucosal T_cons_ by in vitro expanded T_regs_ is currently unknown and will be the subject of further study. In addition, not all of the patients in this study had active disease, so it will be important to extend these data further to broaden the therapeutic relevance of these findings. However, a substantial proportion of the patients in this study did have evidence of disease activity (n=5/13), which did not affect either T_reg_ expansion or function.

In conclusion, we have shown that in vitro expanded CD45RA^+^ T_regs_ are likely to be the most suitable T_reg_ subset for cellular therapeutics in CD. This subset is readily expandable to sufficiently high numbers under conditions that are readily transferable to GMP, for clinical use. They express an appropriate repertoire of homing receptors for MLN and gut, and effectively traffic to inflamed gut in vivo. As well as retaining powerful suppressive properties, these cells show little or no capacity for plasticity towards a potentially harmful effector phenotype, which correlates with an epigenetically stable *FOXP3* locus. This study addresses many of the perceived barriers to T_reg_ cell treatment for CD and paves the way for a clinical trial of in vitro expanded CD45RA^+^ T_regs_ in this therapeutically challenging disease.

## Supplementary Material

Web supplement

Web figures
