# Supplementary material for: Developing in vitro expanded CD45RA+ regulatory T cells as an adoptive cell therapy for Crohn's disease
Source: Gut. 2015 Feb 24;65(4):584–94. doi: 10.1136/gutjnl-2014-306919 (PMC4819603; doi:10.1136/gutjnl-2014-306919)
Supplement: Web supplement [file gutjnl-2014-306919-s1.pdf]

Supplemental Methods

In vitro generation of T_{reg} lines

T_{regs} were expanded *in vitro* in X-VIVO-15 (Lonza, Walkersville, MD), supplemented with 5% human AB serum (Biosera, Uckfield, UK) and rapamycin (100nM, Rapamune, Pfizer, Sandwich, UK). Sorted precursor T_{regs} were plated at 10^6/ml and stimulated with anti-CD3/anti-CD28-coated beads (Dynalbeads, Invitrogen, Paisley, UK) at a 1:1 ratio, then incubated at 37°C/5% CO_2. rhIL-2 (1,000IU/ml, Proleukin®, Novartis, Basel, Switzerland) was added at the start of culture and replenished every other day. Re-stimulation frequency was optimized by daily assessment of proliferation and Ki-67 expression. Cells were re-stimulated with new beads after 10-12 days of culture, with the addition of fresh rapamycin and IL-2. The phenotype and suppressive ability of T_{reg} lines was assessed after 22-24 days of culture.

Other experiments were performed in RPMI 1640 (PAA) supplemented with HEPES (10mM, Thermo Fisher Scientific, Loughborough, UK), L-glutamine (2mM), penicillin (100IU/ml), streptomycin (100g/ml), sodium pyruvate (1mM), MEM nonessential amino acids (0.1mM), and 10% fetal calf serum (all PAA).

Antibodies for Flow Cytometry

Live/Dead Aqua staining was performed for 20 minutes at room temperature. Cell surface staining was performed for 30 minutes at 4°C. Intracellular staining was performed using the "FoxP3/Transcription Factor Staining Buffer Set" (eBioscience, Hatfield, UK). Isotype and "fluorochrome

minus one" controls were acquired, as appropriate. Experiments were acquired on LSRII and Fortessa SORP cytometers running FACSDiva 6.1.3 software (BD), and analysed with FlowJo v9.8 for Mac (FlowJo, Ashland OR, USA).

The following antibodies used for flow cytometry: CD3-APC-H7 (clone SK7), CD4-FITC (SK3), CD4-V500 (RPT-T4), CD8-V500 (RPA-T8), CD8-PE-Cy7 (RPA-T8), CD25-PE (2A3), CD45RO-PE-Cy7 (UCHL1), CD154-APC (89-76), CCR5-PE-CF594 (2D7/CCR5), CCR7-PE-CF594 (150503), CXCR3-PE-Cy7 (IC6/CXCR3), and IL-17-Pacific Blue (N49-653, all BD Biosciences); CD3-Pacific Blue (OKT3), CD4-eFlour450 (OKT4), anti-mouse CD45-PerCP-Cy5.5 (30-F11), CD127-PerCP-Cy5.5 (eBioRDR5), FOXP3-FITC (PCH101, all eBioscience); α_4 integrin/CD49d-PerCP-Cy5.5 (9F10), β_7 integrin-FITC (FIB504), CD45RA-AlexaFluor700 (HI100), CD62L-Brilliant Violet (BV) 421 (DREG-56), CD127-BV650 (A019D5), CD161-BV605 (HP-3G10), CCR4-PE-Cy7 (TG6/CCR4), CCR6-APC (G034E3), CCR9-Alexa Fluor 647 (BL/CCR9, all BioLegend). Dead cells were excluded using Live/Dead Blue (Invitrogen) or propidium iodide (Sigma).

Assessment of the in vitro suppressive ability of putative Tregs

FACS-sorted $CD4^+CD25^-$ PB T_{cons}, or LPMCs or MLN mononuclear cells were used as responder cells, as indicated in the text.

In order to assess the ability of putative T_{regs} to suppress proliferation of responder cells *in vitro*, $5x10^4$ responder cells were labelled with CellTrace Violet (CTV, 1μM, Invitrogen) and cultured alone (a 1:0 responder cell:T_{reg} ratio), or with T_{regs} at responder cell:T_{reg} ratios ranging from 1:1 to 32:1 for 96h. Responder cell numbers were kept constant. $CD4^+CD25^-$ T_{cons} were stimulated

with Dynalbeads at a T_{con}:bead ratio of 40:1. LPMCs and MLNMCs were stimulated with plate-bound anti-CD3 and anti-CD28 antibodies (R&D Systems, Abingdon, UK; coated with 2μg/ml of each in PBS at 4°C overnight). Controls included a "2X control" condition (2:0 responder cell:T_{reg} ratio; to exclude non-specific cell density-mediated inhibition of proliferation), and a stained-unstimulated control. The number of non-proliferating cells and the number of precursors of proliferating cells were calculated using standard formulas[1]. Percent suppression of proliferation (*S*) was calculated using the following formula:

$$S = 100 - (\left[\frac{a}{b}\right] x100)$$

where *a* is the percentage of proliferating precursors in the presence of T_{regs} and *b* is the percentage of proliferating precursors in the absence of T_{regs}.

The BD Fastimmune Human Regulatory T Cell Function Kit (BD Biosciences) was used according to manufacturer's instructions to assess the ability of putative T_{regs} to suppress activation of LPMCs and MLNMCs *in vitro*. Briefly, responder cells were cultured for 7h at the cell numbers and responder cell:T_{reg} ratios described above. Cells were stimulated with plate-bound anti-CD3 and anti-CD28, as described above. And anti-CD154 (CD40L)-APC (clone 89-76) was added at time 0. Controls included a "2X control", unstained-unstimulated, stained-unstimulated and stained-unstimulated responder cell conditions. Assessment of T_{reg}-mediated suppression of CD154 expression on lymphocytes was performed as described previously[2,3]. Percent suppression of proliferation (*S*) was calculated using the following formula:

$$S = 100 - (\left[\frac{c}{d}\right] x100)$$

where c is the percentage of $CD154^{+}$ events in live $CD3^{+}$ responder cells in the presence of Tregs, while d is the percentage of $CD154^{+}$ events in live $CD3^{+}$ responder cells in the absence of T_{regs}.

rtPCR

The following FAM-conjugated probes were used: AHR (Hs00169233), CCR9 (Hs01890924), IL-17A (Hs00174383) and RORC (Hs01076112). GAPDH-VIC (Applied Biosystems, Paisley, UK) was used as an endogenous housekeeping gene.

Isolation of LPMCs and MLN mononuclear cells

LPMCs were isolated from inflamed mucosa and peri-colic fat obtained from CD right hemicolectomy resection specimens. Mucosal epithelium was removed by agitation in pre-warmed HBSS (PAA) supplemented with EDTA (1 mM, Sigma) and gentamycin (30 mg/ml, PAA) for 30 minutes at 37°C. A single cell suspension was then prepared by agitation with pre-warmed RPMI supplemented with collagenase 1a (1 mg/ml, Sigma), DNase I (10 IU/ml, Roche) and gentamycin for 60 minutes at 37°C. LPMCs were then enriched by gradient density centrifugation.

MLNs were harvested from peri-colic fat of the same resections. MLNs were mechanically disrupted between mesh, then washed with cold RPMI, to yield a single layer suspension of MLN mononuclear cells.

C.B-17 SCID mouse human intestinal xeno-transplant model

IRB and IACUC approvals were obtained prospectively (Ethics Committee for Animal Experimentation, Hebrew University of Jerusalem; MD-11-12692-4 and the Helsinki Committee of the Hadassah University Hospital; 81-23/04/04). Women undergoing legal terminations of pregnancy gave written, informed consent for use of fetal tissue in this study.

C.B-17 SCID mice were purchased from Harlan, Israel and housed under SPF conditions. Human fetal small bowel up to 16 weeks gestational age was implanted subcutaneously on the dorsum of the mouse, as described previously[4,5]. Grafts developed *in situ* for 12-16 weeks prior to manipulation. Mice were treated IP with rabbit anti-asialo GM1 (15μl, Cedarlane Labs, Burlington NC) 24h prior to T_{reg} administration. As murine IL-2 is significantly less efficient at promoting the survival and proliferation of human T cells than human IL-2[6], mice were treated with rhIL-2 ($2x10^4$IU, Proleukin) directly prior to T_{reg} administration, as described by Tresoldi *et al.*[7]. $10x10^6$ T_{regs} that were expanded *in vitro* from $CD4^+CD25^{hi}CD127^{lo}CD45RA^+$ precursors were administered *via* tail vein injection. Homing was assessed 24h later.

Enteropathogenic *Escherichia coli* (EPEC) WT-GFP were grown in LB medium overnight at 27°C without agitation, as described previously[5]. In order to induce mucosal inflammation, up to 10^8 bacteria in 100μl PBS (or PBS alone negative control) was injected percutaneously into the lumen of the human small intestinal grafts. were injected intraluminally with either up to 10^8 bacteria

in 100μl PBS or PBS alone. Because mice could accommodate 2 xenografts, each animal acted as their own control.

Twenty four hours after IV adoptive transfer of T_{regs}, animals were sacrificed and T_{regs} homing to the human small intestinal grafts was assessed by flow cytometry and immunofluorescence.

In order to detect adoptively transferred T_{regs} by FACS, single cell suspensions were prepared from murine spleen and xenograft LP, as described above, followed by staining with anti-mouse CD45-PerCP-Cy5.5 (30-F11), anti-human CD45-Pacific Blue (2D1), anti-human CD4-FITC (OKT4, all eBioscience), anti-human CD3-APC-H7 (SK7, BD) and live/dead blue, then FACS acquisition.

In order to detect adoptively transferred T_{regs} by immunofluorescence, fixed cryostat sections were blocked with 20% horse serum (PAA) and stained with anti-human CD45-FITC (2D1) and anti-human CD3-biotin (OKT3, both eBioscience), followed by streptavidin-AlexaFluor 594 (Invitrogen). To visualize EPEC, sections were stained with phalloidin-rhodamine (Sigma). Nuclei were stained with DAPI (1g/ml, Invitrogen). Negative controls were stained with isotype-matched antibodies. Images were acquired on an Olympus BX51 microscope using Micro-Manager software (Vale Lab, UCSF, San Francisco, CA).

Statistical analysis

The Kolmogorov-Smirnov test was used to determine if continuous variables were normally distributed. Normally distributed variables were then compared using unpaired or paired t tests. Non-normally distributed variables were

compared using Mann-Whitney U or Wilcoxon matched-pairs signed rank tests, as appropriate. Paired tests were used with paired data. Categorical variables were compared using Chi-square tests. Grouped variables were compared using ANOVA. Post-hoc corrections were not performed. A p value of <0.05 was considered statistically significant.

Supplement references

1 Wells AD, Gudmundsdottir H, Turka LA. Following the fate of individual T cells throughout activation and clonal expansion. Signals from T cell receptor and CD28 differentially regulate the induction and duration of a proliferative response. *J Clin Invest* 1997;**100**:3173–83. doi:10.1172/JCI119873

2 Canavan JB, Afzali B, Scottà C, *et al.* A rapid diagnostic test for human regulatory T-cell function to enable regulatory T-cell therapy. *Blood* 2012;**119**:e57–66. doi:10.1182/blood-2011-09-380048

3 Canavan JB, Afzali B, Lord GM, *et al.* Assessment of regulatory T-cell function in forthcoming clinical trials of cell therapy. *Expert Rev Mol Diagn* 2013;**13**:5–7. doi:10.1586/erm.12.133

4 Howie D, Spencer J, DeLord D, *et al.* Extrathymic T cell differentiation in the human intestine early in life. *J Immunol* 1998;**161**:5862–72.

5 Golan L, Gonen E, Yagel S, *et al.* Enterohemorrhagic *Escherichia coli* induce attaching and effacing lesions and hemorrhagic colitis in human and bovine intestinal xenograft models. *Dis Models Mech* 2010;**4**:86–94. doi:10.1242/dmm.005777

6 Mosmann TR, Yokota T, Kastelein R, *et al.* Species-specificity of T cell stimulating activities of IL 2 and BSF-1 (IL 4): comparison of normal and recombinant, mouse and human IL 2 and BSF-1 (IL 4). *J Immunol* 1987;**138**:1813–6.

7 Tresoldi E, Dell'Albani I, Stabilini A, *et al.* Stability of human rapamycin-expanded CD4+CD25+ T regulatory cells. *Haematologica* 2011;**96**:1357–65. doi:10.3324/haematol.2011.041483
