# Supplementary material for: Developing in vitro expanded CD45RA+ regulatory T cells as an adoptive cell therapy for Crohn's disease
Source: Gut. 2015 Feb 24;65(4):584–94. doi: 10.1136/gutjnl-2014-306919 (PMC4819603; doi:10.1136/gutjnl-2014-306919)

# *Supplemental Figures*

**Figure S1. Strategy to determine the optimum enrichment method for *in vitro* expansion of $T_{regs}$ from PB of CD patients.**

(A-E) Cartoon illustrating the strategy used to enrich starting populations for $T_{reg}$ expansion. (A) PB was obtained from CD patients and (B) PBMCs enriched by density gradient centrifugation. (C) Cells for culture were then enriched with MACS technology using two strategies achievable with currently available reagents for the Miltenyi "CliniMACS" system. (D) PBMCs were enriched in a two-step selection involving $CD8^+$ depletion, followed by $CD25^+$ enrichment. (D') Alternatively, PBMCs were positively selected for $CD4^+$, then stained for CD4, CD25, CD127 and CD45RA, (D'') followed by sorting on a BD FACSAria II into $CD4^+CD25^{hi}CD127^{lo}CD45RA^+$ and $CD4^+CD25^{hi}CD127^{lo}CD45RA^-$ subsets. (E) Each enriched population was stimulated with anti-CD3/anti-CD28 coated Dynabeads and cultured for 20-24 days in X-VIVO15 supplemented with 5% human AB serum, IL-2 and rapamycin. ILX2 was replenished every other day. Cells were re- stimulated and placed in fresh medium at day 12.

Figure S1

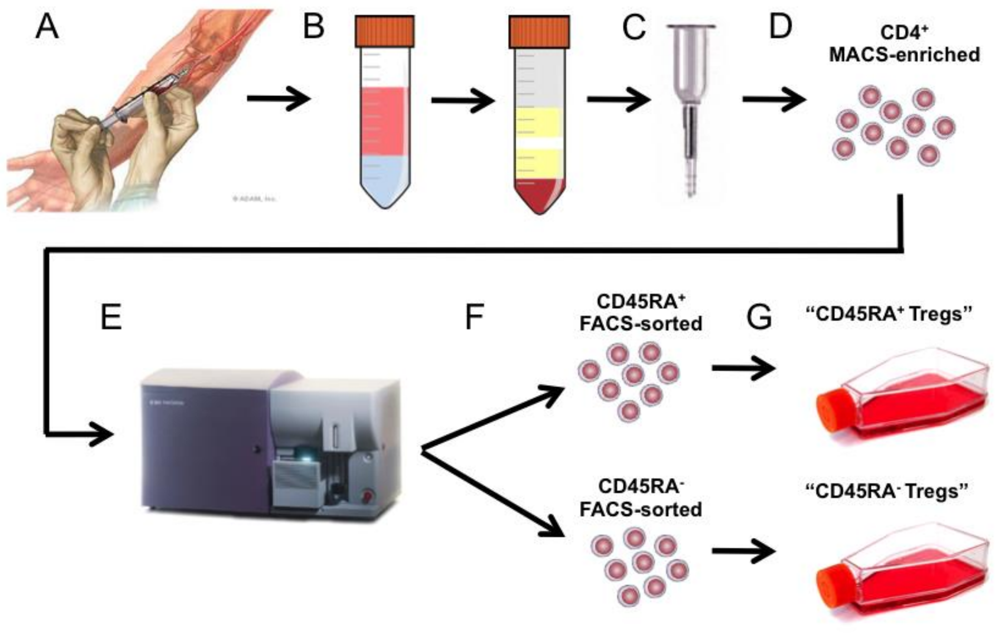


**Figure S2. Gating strategy for FACS sorting of MACS-enriched CD4$^+$ lymphocytes into CD4$^+$CD25$^+$CD127$^{lo}$CD45RA$^+$ and CD4$^+$CD25$^+$CD127$^{lo}$CD45RA$^-$ subsets for subsequent expansion *in vitro*.**

(A) Pre-sort CD4$^+$ lymphocytes. Following exclusion of doublets, lymphocytes are sorted on the basis of CD4$^+$CD25$^{hi}$, then CD127$^{lo}$, then CD45RA expression. (B) Post-sort enrichment for CD4$^+$CD25$^+$CD127$^{lo}$CD45RA$^+$ $T_{regs}$. (C) Post-sort enrichment for CD4$^+$CD25$^+$CD127$^{lo}$CD45RA$^-$ $T_{regs}$. (D) Post-sort enrichment for autologous CD4$^+$CD25$^-$ $T_{cons}$. Representative of 13 independent experiments. (E) Cell counts per ml of PB according to disease activity status and sorted $T_{reg}$ subset.

Supplemental Figure 2

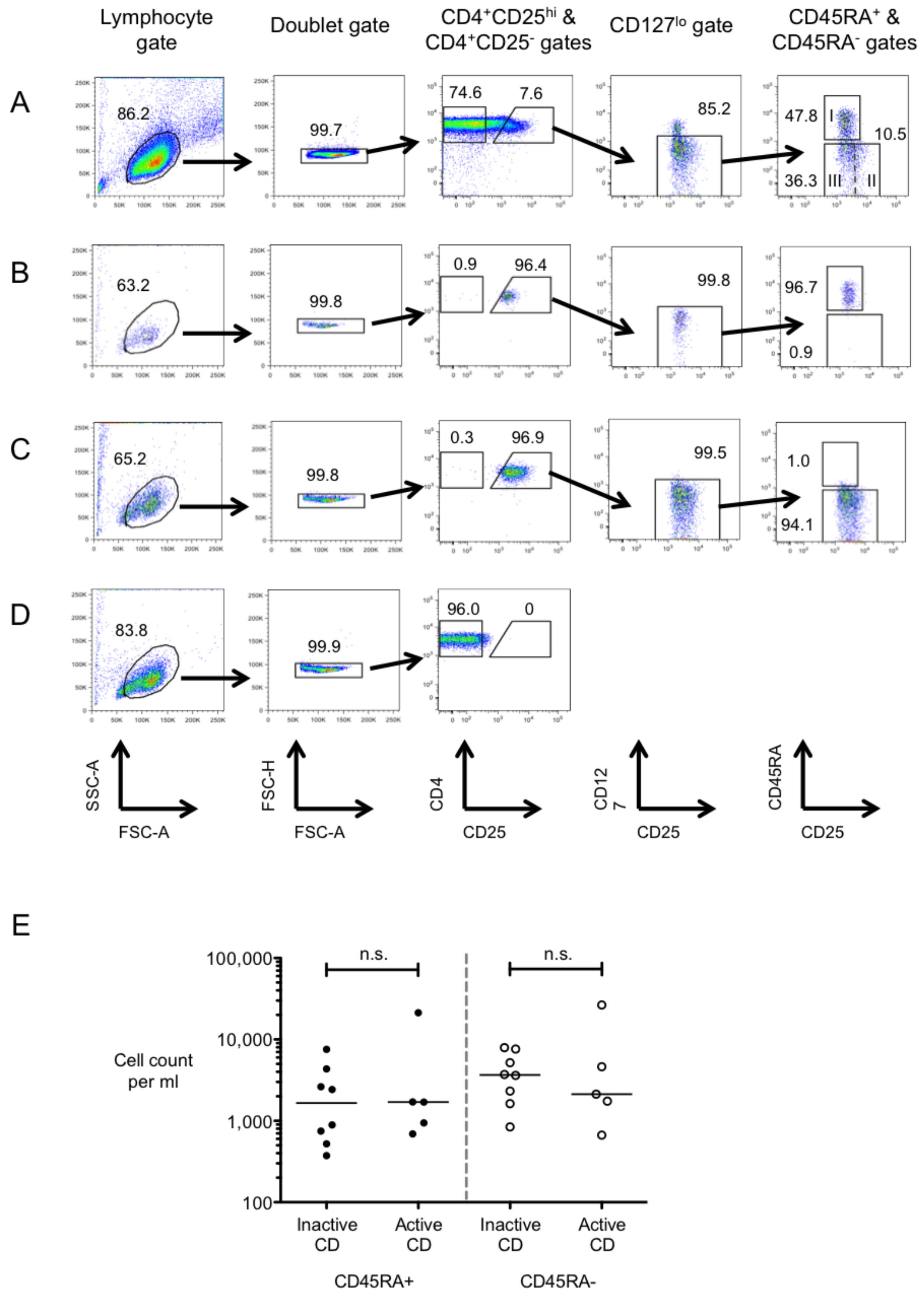

**Figure S3. $T_{reg}$-mediated reduction of IL-2 and IFN-γ in 96h co-culture supernatants.**

(A) $T_{reg}$-mediated reduction of IL-2 in supernatants correlated with $T_{reg}$-mediated suppression of proliferation for both CD45RA$^{+}$ (left panel) and CD45RA$^{-}$ $T_{regs}$ (right panel). (B) $T_{reg}$-mediated reduction of IFN-γ in supernatants correlated with suppression of proliferation for CD45RA$^{+}$ $T_{regs}$ (left panel) but not CD45RA$^{-}$ $T_{regs}$ (right panel).

Supplemental Figure 3

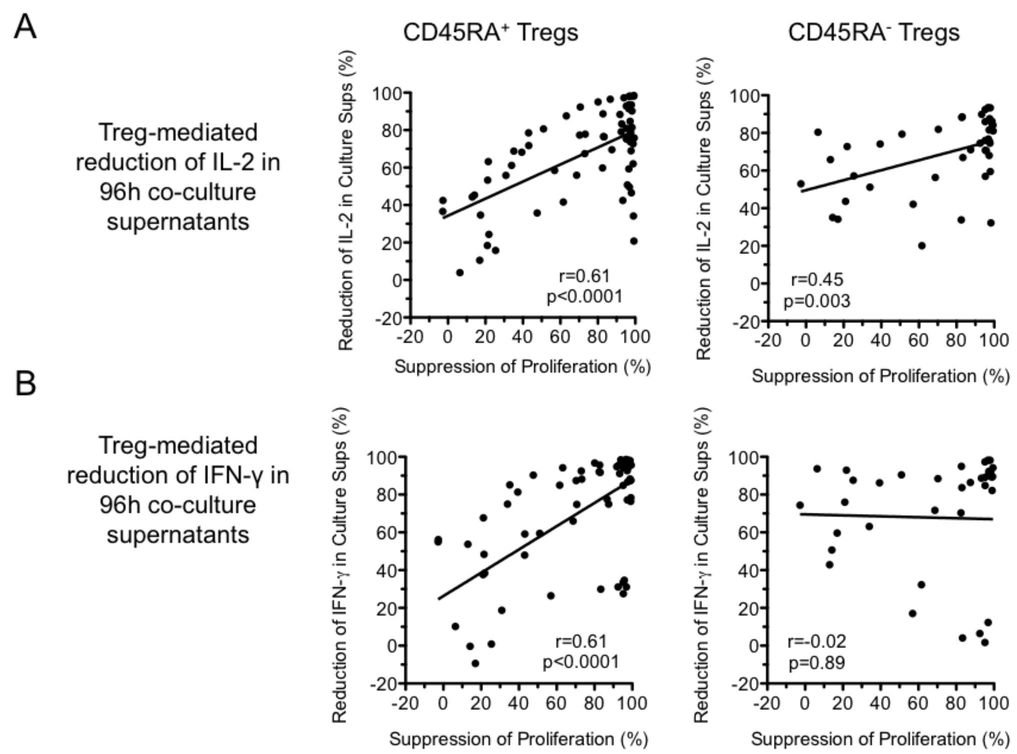

**Figure S4. C.B-17 SCID intestinal xenograft model.**

(A) EPEC induces mucosal inflammation in human fetal intestinal xeno-transplants. Top left panel: Representative microscopic image of a xenograft 8h after intraluminal injection with PBS, showing non-inflamed small bowel mucosa with normal villi (arrows) and intestinal crypts (arrowheads). H&E, 20X. Bottom left panel: In contrast, intraluminal injection with EPEC caused mucosal inflammation with destruction of villi and cytoplasmic vacuolization. Top right panel: Fluorescence staining of PBS-treated xenograft cryosection showing a normal small bowel villus. F-Actin is visualized with phalloidin-rhodamine (red). DAPI (blue). Bottom right panel: GFP-expressing EPEC (green, arrows) adhere to mucosal epithelial cells and induce formation of AE lesions. (B) FACS plots showing healthy control PBMCs and the gating strategy for identification of live human $CD45^{+}CD3^{+}CD4^{+}$ events in the C.B17 SCID human intestinal xeno-transplant model.

Supplemental Figure 4

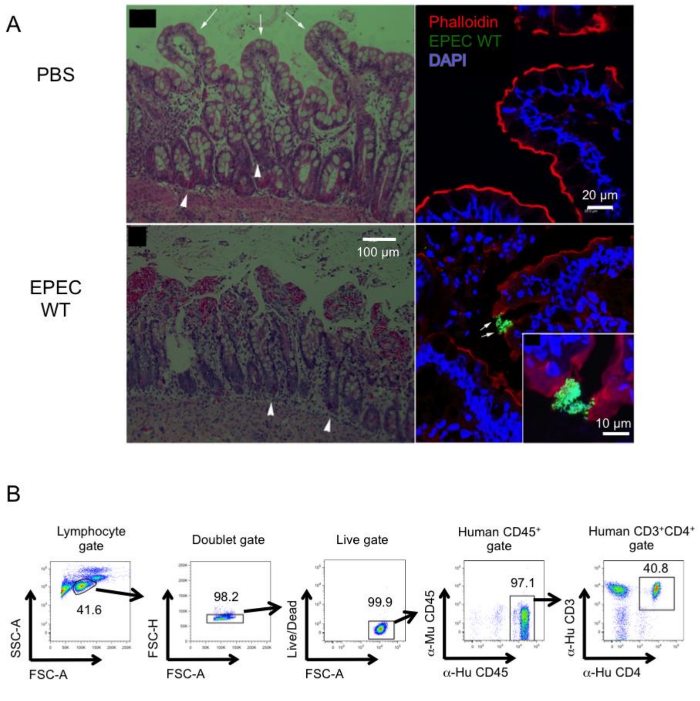

**Figure S5. Viability and proliferation of healthy donor PBMCs following 5 days' *in vitro* proliferation.**

(A) Representative microscopic image of thawed healthy donor PBMCs showing good viability (0.4% Trypan blue, 100X). (B) PBMCs were then labelled with CFSE or CellTrace Violet (CTV) and cultured for 5 days, stimulated with plate-bound anti-CD3/anti-CD28 antibodies. End-of-culture representative forward- *vs.* side-scatter plot is shown. (C) Representative plots illustrating live events in CFSE-labelled (left) and CTV-labelled (right) PBMCs, at 5 days. (D) Representative plots illustrating proliferation at 5 days.

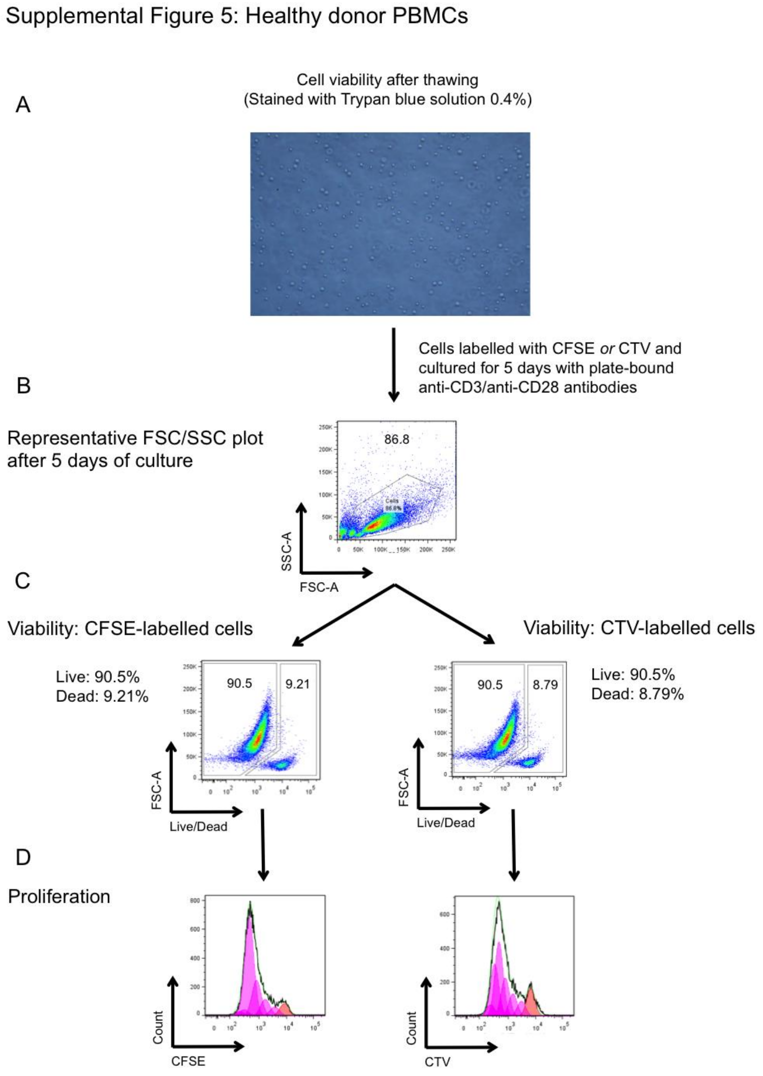

**Figure S6. Viability and proliferation of CD LPMCs following 5 days' *in vitro* proliferation.**

(A) Representative microscopic image of thawed CD LPMCs showing poor viability and clumping (0.4% Trypan blue, 100X). (B) LPMCs were then treated as described in Figure S4B. End-of-culture representative forward- *vs.* side-scatter plot is shown. (C) Representative plots illustrating live events in CFSE-labelled (left) and CTV-labelled (right) LPMCs, at 5 days. Similar results were obtained using fresh or thawed LPMCs. (D) Poor viability at 5 days meant that assessment of proliferation was not feasible.

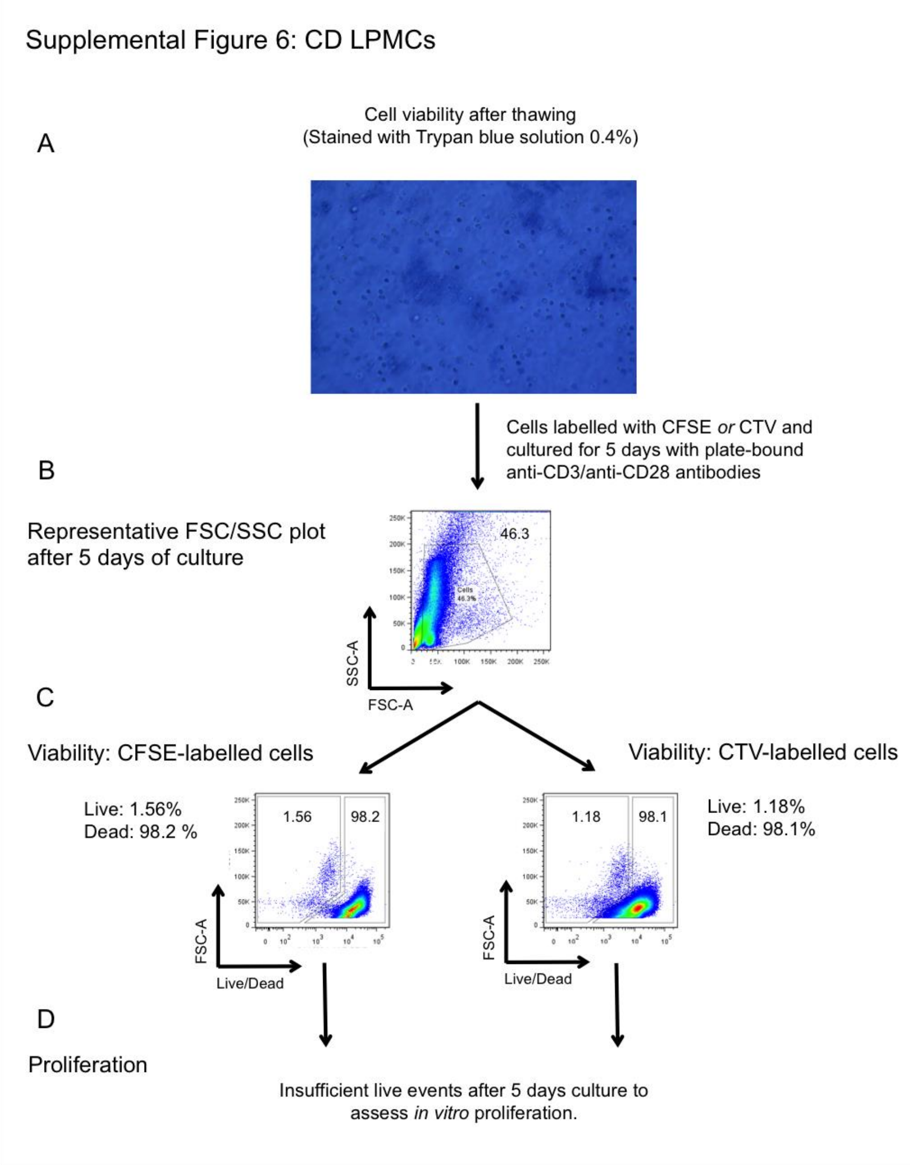

Supplement: Web figures [file gutjnl-2014-306919-s2.pdf]
